# Phylogenetic Group/Subgroups Distributions, Virulence Factors, and Antimicrobial Susceptibility of *Escherichia coli* Strains from Urinary Tract Infections in Hatay

**DOI:** 10.1590/0037-8682-0429-2019

**Published:** 2020-02-07

**Authors:** Ebru Şebnem Yılmaz, Özkan Aslantaş

**Affiliations:** 1 Department of Biology, Faculty of Art and Science, Hatay Mustafa Kemal University, TR-31060 Hatay, Turkey.; 2 Department of Microbiology, Faculty of Veterinary Medicine, Hatay Mustafa Kemal University, TR-31060 Hatay, Turkey.

**Keywords:** Urinary Tract Infections, Virulence genes, Phylogenetic group, Antimicrobial resistance

## Abstract

**INTRODUCTION:**

Nosocomial and community acquired urinary tract infections (UTIs) are one of the most encountered infections in the world.

**METHODS:**

This study aimed to determine the antibiotic susceptibility, phylogeny, and virulence genes of 153 *Escherichia coli* strains isolated from UTIs. Antimicrobial susceptibility of the isolates to different classes of antimicrobials was determined by the VITEK-2 automated system. Presence of virulence genes and phylogenetic groups were investigated by PCR.

**RESULTS:**

Regarding susceptibility to antimicrobials, ampicillin resistance was most abundant (67.3%), followed by amoxicillin-clavulanic acid (50.9%); least abundant was resistance to amikacin (1.3%) and nitrofurantoin (1.3%). Multi drug resistance (MDR) was observed in 34.6% of the isolates, and all isolates were found to be susceptible to imipenem, meropenem and fosfomycine. The majority of the isolates belonged to the phylogenetic group B2_3_ (35.9%), followed by A1 (20.9%), D1 (18.9%), D2 (12.4%), A0 (%5.9), B1 (3.9%) and B2 (1.9%). Among *E. coli* strains examined, 49% had *iuc*D, 32.7% *pap*E-F, 26.1% *pap*C, 15% *cnf*2, 11.1% *sfa*, 7.8% *cnf*1, 1.3% *afa*E, 1.3% *afa*D, 1.3% *hly*A, 0.7% *f17a*-A, 0.7% *clp*G and 0.7% *eae*A genes.

**CONCLUSIONS:**

Our research demonstrated that virulence factors were distributed among different phylogroup/subgroups, which play a role in UTIs pathogenesis in humans. For this reason, complex and detailed studies are required to determine the relationship between virulence factors and specific *E. coli* strains that cause UTIs in humans.

## INTRODUCTION

Urinary tract infections (UTIs) are one of the most common infections, affecting both outpatients and inpatients worldwide^1^. Uropathogenic *Escherichia coli* (UPEC), classified as Extraintestinal pathogenic *E. coli* (ExPEC), are one of the most predominant causes of UTIs^2^. UPEC strains have several virulence factors which play an important role in the pathogenesis of infections. These virulence factors include both structural (fimbriae, pili, curli, flagella) and secreted (toxins, iron-acquisition) systems^3^, are related to colonization and durability of bacteria in the urinary system^4^. In addition, it has been shown that *E. coli* strains causing UTIs have a higher prevalence rate of virulence genes than commensal *E. coli* strains^5^.

Based on three genetic marker, including *chu*A, *yja*A and DNA fragment TSPE4.C2, *E. coli* strains were mainly divided into four phylogenetic groups (A, B1, B2 and D) by Clermont et al. (2000)^6^. Escobar-Páramo et al. (2004) further divided these phylogenetic groups into subgroups according to presence or absence of the *chu*A, *yja*A genes and the DNA fragment TSPE4.C2 including A0, A1, B1, B2, B2_3_, D1, D2^7^. Previous phylogenetic analysis revealed that ExPEC strains causing UTIs mainly belonged to phylogenetic groups B2 or D, but commensal strains predominantly belonged to phylogenetic groups B1 or A^4^ ^,^ ^8^. Phylogenetic grouping of *E. coli* isolates is of importance not only for understanding of *E. coli* populations, but also elucidating the relationship between strains and disease.

As observed in other bacterial pathogens, increasing antimicrobial resistance in ExPEC strains poses a serious public health threat by decreasing available treatment options for UTIs. Therefore, continuous surveillance of ExPEC strains for antimicrobial susceptibility may provide useful information that will assist physicians in administering effective UTI treatment^9^.

Previously, there have been a few studies featuring virulence properties, antibiotic resistance, and its relationship with phylogenetic groups among *E. coli* associated with UTIs in Turkey^10^ ^-^ ^13^. Therefore, the main objective of this study was to determine the antimicrobial susceptibility, phylogeny, and virulence genes of *E. coli* isolated from patients admitted to Hatay State Hospital with UTI complaint.

## METHODS

*E. coli* strains were isolated from urine samples collected from patients admitted to Antakya State Hospital with complaint of UTI between January and June 2014. Isolates were included in the study when a pure culture containing > 10^5^ cfu/ml was acquired. The isolates were identified with conventional biochemical tests^14^ (Gram staining, oxidase, IMVIC), and confirmed by polymerase chain reaction (PCR) targeting *E. coli* specific 16S rRNA^15^.

Antimicrobial susceptibility of the isolates were performed using an automated method (VITEK^®^2 BioMérieux). Susceptibility to 17 antimicrobials including ampicillin, amikacin, amoxycillin-clavulanic acid, cefazolin, cefepime, cefoxitin, ceftriaxone, cefuroxime, ciprofloxacin, fosfomycin, gentamicin, imipenem, meropenem, nitrofurantoin, norfloxacin, trimethoprim*-*sulfamethoxazole and piperacillin-tazobactam was tested using a Gram Negative Susceptibility card (AST-N325). The isolates showing resistance to three or more antimicrobials from different classes of antimicrobials were categorized as multi drug resistant (MDR).

Bacterial genomic DNA was acquired by boil extraction method^16^. Phylogenetic grouping of the isolates was determined using multiplex PCR^6^. The identification of phylogenetic groups and subgroups (A0, A1, B1, B2, B2_3_, D1, D2) were determined based on presence or absence of the *chu*A, *yja*A genes and the DNA fragment TspE4-C2 as previously described by Escobar-Páramo et al^7^. 

The frequency of virulence genes (*pap*C, *pap*E-F, *sfa/foc*DE*, cnf1, iuc*D, *hly*A, *afa D-8*, *afa E-8*, *clp*G, *cnf*2, *f17*A, *f17a-*A, *f17b-*A, *f17c-*A, *f17d-*A, *stx*1, *stx*2, and *eae*A) were investigated using PCR protocols^15^ ^,^ ^17^ ^-^ ^21^.

Statistical differences among phylogenetic groups/subgroups, virulence genes, and antimicrobial susceptibility results were determined using Pearson’s chi-square test. SPSS 14.01 was used for statistical analysis. In all statistical analyses a level of significance of 0.05 was adopted.

## RESULTS

**FIGURE 1:**
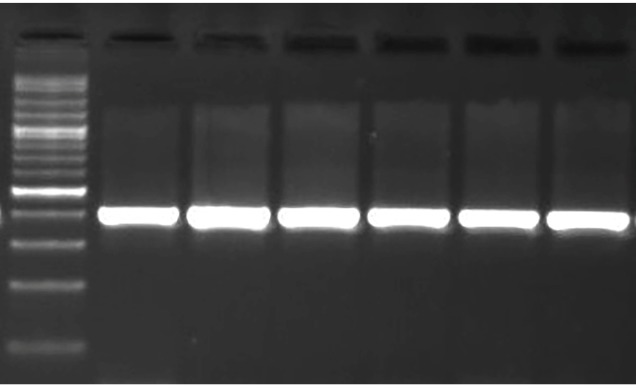
PCR amplification of *E. coli* specific 16S rRNA gene (401 bp).

A total of 153 strains isolated from patient urine specimens were identified as *E. coli* based on standard biochemical tests and PCR amplification of the targeted 16S rRNA (Figure 1).

**FIGURE 2:**
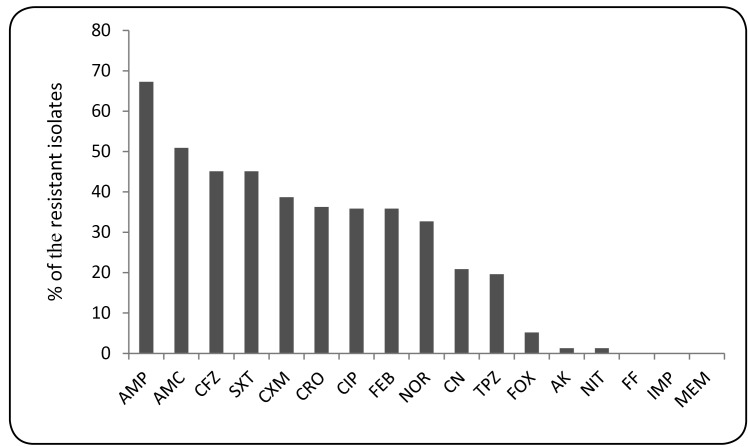
Antimicrobial resistance rates of *E. coli* isolates. **AMP:** Ampicillin; **AMC:** Amoxycillin-clavulanic acid; **CFZ:** Cefazolin; **SXT:** Trimethoprim-sulfamethoxazole; **CXM:** Cefuroxime; **CRO:** Ceftriaxone; **CIP:** Ciprofloxacin; **FEB:** Cefepime; **NOR:** Norfl oxacin; **CN:** Gentamicin; **TPZ:** Piperacillin-tazobactam; **FOX:** Cefoxitin; **AK:** Amikacin; **NIT:** Nitrofurantoin; **FF:** Fosfomycin; **IMP:** Imipenem; **MEM:** Meropenem.

Antimicrobial susceptibility testing revealed that all isolates were susceptible to imipenem, meropenem and fosfomycine. Various rates of resistance to ampicillin (67.3%, n=103), amoxicillin-clavulanic acid (50.9%; n=78), cefazolin (45.1%, n=69), trimethoprim-sulfamethoxazole (45.1%, n=69), cefuroxime (38.65%, n=59), ceftriaxone (36.6%, n=56), ciprofloxacin (35.9%, n=55), cefepime (35.9%, n=55), cefoxitin (5.2%, n=8), norfloxacin (32.7%, n=50), gentamicin (20.9%, n=36), tazobactam-ticarbenicillin (19.6%; n=30), amikacin (1.3%, n=2) and nitrofurantoin (1.3%, n=2) were observed (Figure 2). MDR was observed in 34.6% (n=53) of the isolates. Forty (26.1%) isolates were found to be susceptible to all antimicrobials tested. There were no statistically significant differences among MDR, non-MDR, and susceptible isolates among phylogenetic groups/subgroups (P>0.672). 

**FIGURE 3:**
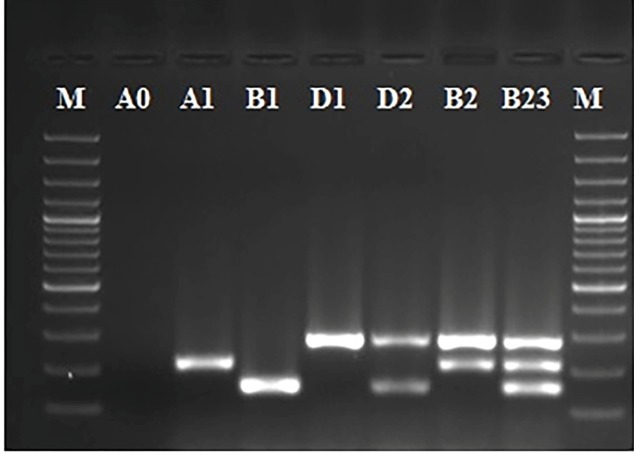
Phylogenetic groups determined among *E. coli* isolates.

Phylogenetic grouping and subgrouping was determined as follows: 55 (35.9%) isolates belonged to group B2_3_, 32 (20.9%) belonged to group A1, 29 (18.9%) belonged to D1, 19 (12.4%) belonged to D2, 9 (5.9 %) belonged to A0, 6 (3.9 % ) belonged to B1, and 3 (1.9 %) belonged to B2 (Figure 3).

Of the 153 *E. coli* isolates, 109 (71.2%) isolates carried at least one virulence gene. Distribution of virulence genes was detected as *iuc*D (49%, n=75), *pap*E-F (32.7%, n=50), *pap*C (26.1%, n=40), *cnf*2 (15%, n=23), *sfa* (11.1%, n=17), *cnf*1 (7.8%, n=12), *afa*E (1.3%, n=2), *afa*D (1.3%, n=2), *hly*A (1.3%, n=2), *f17a*-A (0.7%, n=1), *clp*G (0.7%, n=1) and *eae*A (0.7%, n=1), respectively. In addition, 26 different virulence gene profiles were observed among the isolates. Distribution of virulence gene profiles based on phylogenetic groups/subgroups among the isolates were given in Table 1. Statistically significant differences were observed between the phylogenetic groups and the isolates with and without the virulence gene (P<0.001).

**TABLE 1:** Distribution of virulence gene profiles according to phylogenetic group/subgroups among *E. coli* isolates.

Virulence Genes	Phylogenetic Group/Subgroup						
	A0	A1	B1	B2	B2_3_	D1	D2
*iuc*D, *pap*C, *cnf*1, *pap*E-F, *sfa*, *cnf*2				1	1		
*pap*C, *pap*E-F*, cnf*-1, *cnf*2, *sfa*					4		
*iuc*D, *pap*C, *pap*E-F, *cnf*1, *cnf*2				1			
*iuc*D, *f17a*-A, *afa*D, *afa*E		1					
*pap*C, *pap*E-F, *cnf*2, *sfa*					3		
*iuc*D, *cnf*-1, *cnf*2, *sfa*					1		
*eae*A, *pap*C, *pap*E-F						1	
*iuc*D, *pap*C, *pap*E-F		1			7	10	1
*iuc*D, *pap*C, *sfa*					1		
*iuc*D, *afa*D, *afa*E	1						
*cnf*-1, *cnf*2*, sfa*					3		
*pap*C, *sfa*, *cnf*2					1		
*pap*C, *pap*E-F		1			2	1	1
*iuc*D, *pap*E-F		3			1	5	
*iuc*D, *cnf*2		1					
*iuc*D, *pap*C					2		
*pap*E-F, *sfa*					1		
*sfa*, *cnf*2					1		
*clp*G, *hly*A				1			
*cnf*2*, hly*A			1				
*cnf2*	1	3	1				
*iuc*D		7	1		24	2	3
*pap*E-F						3	2
*f17*A		1					
*pap*C							1
*cnf-*1			1				
Negative	7	14	2		3	7	11
**Total**	**9**	**32**	6	3	**55**	**29**	**19**

## DISCUSSION

Determination of antimicrobial resistance and virulence properties of *E. coli* strains isolated from UTIs are of importance, especially in hospitalized patients, allowing physicians to provide alternative treatment options, reducing the risk of complications, and optimizing ongoing infection control programs^9^. Because UTIs are often treated empirically by physicians, it is therefore necessary to understand the epidemiological data related to agents causing infection in order to improve patient outcomes^22^.

Increased antimicrobial resistance rates, particularly for beta-lactams, sulfamethoxazole-trimethoprim, third generation cephalosporins, and fluoroquinolones, has led to challenges in clinical practice^23^. In this study, nearly half of the isolates were resistant to the majority of the tested antimicrobials, with 34.6% of the strains demonstrating MDR, which is in agreement with previous studies conducted in different regions of Turkey^12^ ^,^ ^24^. In accordance with the results of the study, 67.3%, 50.9%, 45.1%, 45.1%, 38.7%, 36.6%, 35.9%, 35.9%, and 32.7% were resistant to ampicillin, amoxicillin-clavulanic acid, cefazolin, trimethoprim-sulfamethoxazole, cefuroxime, ceftriaxone, ciprofloxacin, cefepime, and norfloxacin, respectively, which are the first-line therapeutic agents used for UTI treatment^23^ ^,^ ^25^. These resistance rates may be explained by the frequent prescription of these antimicrobials in empirical treatment of UTIs.

UPEC strains have numerous virulence factors that enable bacteria to colonize the urinary tract and overcome various host defense mechanisms^26^ ^,^ ^27^. In this study, 28.8% of the isolates were negative for examined genes. On the other hand, 71.2% of the isolates were positive for at least one of the virulence genes examined. Of these virulence factors, adhesion molecules have an important role in the promotion of colonization, invasion, and replication within uroepithelial cells^26^. In this study, the most prevalent adhesion genes were *pap*E-F (32.7%, n:50) and *pap*C (26.1%, n: 40), followed by *sfa* (11.1%, n:17), *afa*E (1.3%, n:2), and *afa*D (1.3%, n:2), respectively. Presence of P fimbria is well documented to be associated with pyelonephritis and cystitis^1^. In a study conducted by Munkhdelger et al. (2017), the frequency of *fim*H, *pap*C, *pap*GII, *afa*/*dra*BC, *sfa*/*foc*DE and *pap*GIII was 89.9%, 20.3%, 17.6%, 15.5%, 8.8% and 1.4% ^28^. In another study conducted in Brazil, Tiba et al. (2008) reported frequency of the virulence genes *fim*H, *pap*C, *sfa*, and *afa* to be 97.5%, 32.7%, 27.8%, and 6.2%, respectively^29^. In Mexico, Paniagua-Contreras et al. (2015) found the prevalence of *fim*, *pap* and *pap*GII as 61.3%, 24.7%, and 21.1%, respectively^1^. The *sfa* gene was found in twelve (70.6 %) of the isolates together with *pap* genes. Shetty et al. (2014) explained that co-existence of these two genes are due to their localization on the same pathogenicity island of UPEC strains^30^. In addition, most of the isolates carried multiple adhesion genes, indicating that the isolates had the ability to adhere to the urinary tract and subsequently cause infection. It has been reported that ExPEC strains mainly belong to groups B2 and D, and have higher virulence genes in relation with isolates considered to be commensal, which belong to the phylogenetic groups A and B1^31^ ^,^ ^32^. Similarly, the phylogenetic groups D1 (29, 18.9%) and B2_3_ (55, 35.9%) were the most common among the isolates carrying virulence genes in the study. In a study carried out in Mexico, Miranda-Estrada et al. (2017) reported that the majority of the isolates belonged to group B2 (60%) and harbored a high number of virulence factors^33^. A similar result was reported in Pakistan by Bashir et al. (2012), who found 50% of UPEC isolates belong to group B2, and to a lesser extent, groups A1 and B1 (19%)^34^. Lee et al. (2015) also reported high prevalence of virulence factors in groups B2 (79.31%) and D (15.51%), followed by groups A (3.44%) and B1 (1.72%) in South Korea^8^. On the other hand, in this study, 26.8% and 3.9% of the isolates were found to belong to the commensal groups A and D. Our results confirmed this hypothesis not only in ExPEC strains, but also in the commensal *E. coli* strains that can cause UTIs^35^ ^,^ ^36^. In addition, Duriez et al. (2001) suggested that the distribution of B1, A and D groups in each population can vary according to various factors (geographic/climatic conditions, dietary factors, the use of antibiotics, host genetic factors) and commensal strains can acquire virulence factors and become potentially pathogenic^37^.

In conclusion, various rates of resistance and virulence factors were determined among the isolates. Therefore, monitoring of *E. coli* isolates should be performed for the effective treatment of UTIs. The results of the study also revealed that *E. coli* isolates from UTIs belong to different phylogroups/subgroups (mainly B2_3_), and harbor single or various virulence gene combinations. For this reason, more detailed studies are needed to determine the relationship between virulence traits and certain *E. coli* clones that cause UTIs in humans.
